# Supplementary material for: Dissection of amino acid acquisition pathways in Borrelia burgdorferi uncovers unique physiological responses
Source: bioRxiv. 2025 Mar 15:2025.03.14.643351. Preprint. [Version 1] doi: 10.1101/2025.03.14.643351 (PMC11952506; doi:10.1101/2025.03.14.643351)
Supplement: Supplement 1 [file media-1.pdf]

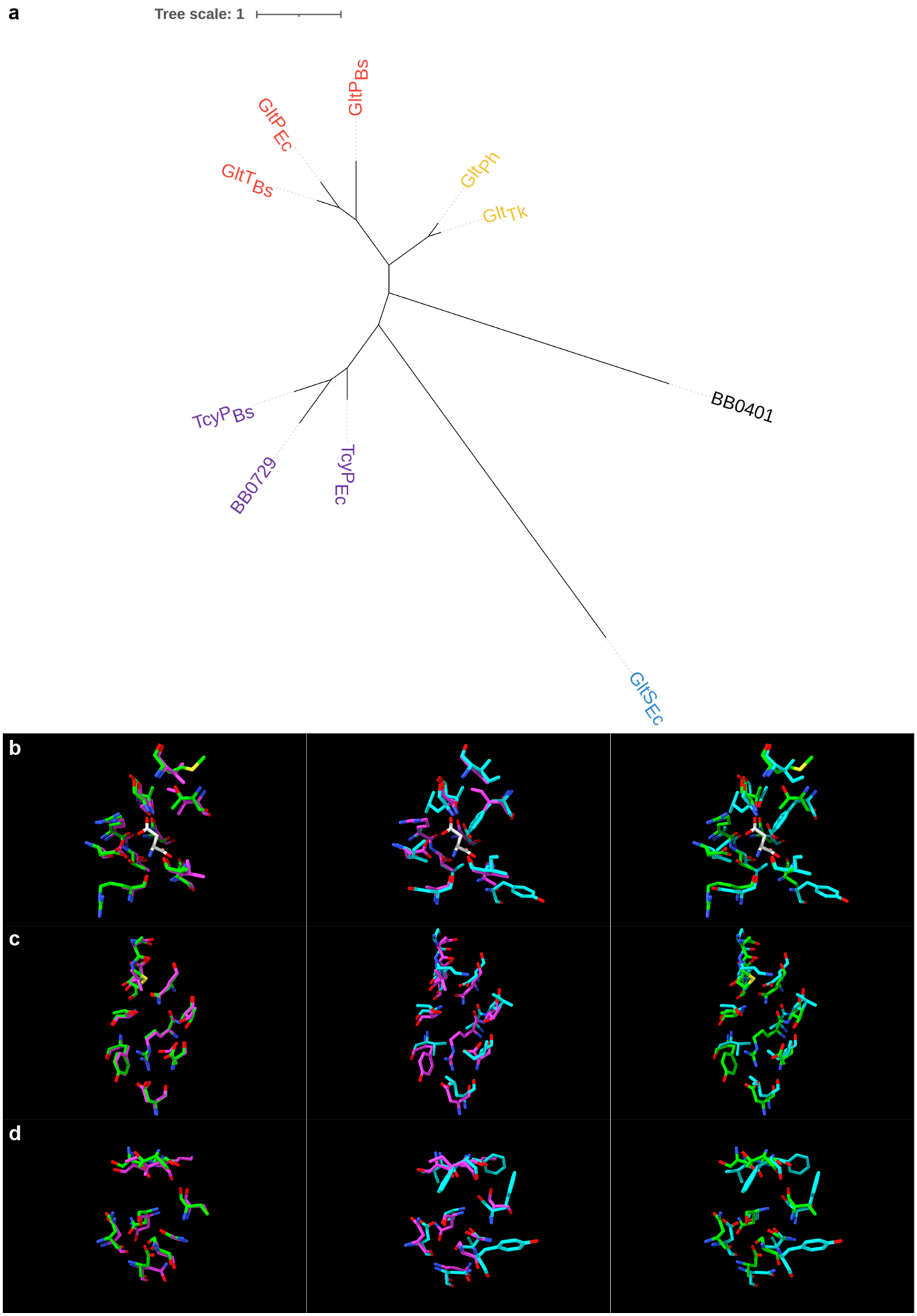

Fig S1: BB0401 models as a GltP. a) Unrooted phylogenetic tree of characterized Glt and TcyP transporters. Dedicated glutamate transporters are blue, aspartate transporters are orange, glutamate and aspartate transporters in red, cystine transporters in purple. b-d) Residue alignments for b) aspartate binding site with Asp from 2nwl, c) predicted glutamate binding site, and c) sodium binding sites. Glt_{Ph} (2nwl) is shown in green, $GltP_{Ec}$ model in magenta, and BB0401 model in cyan.

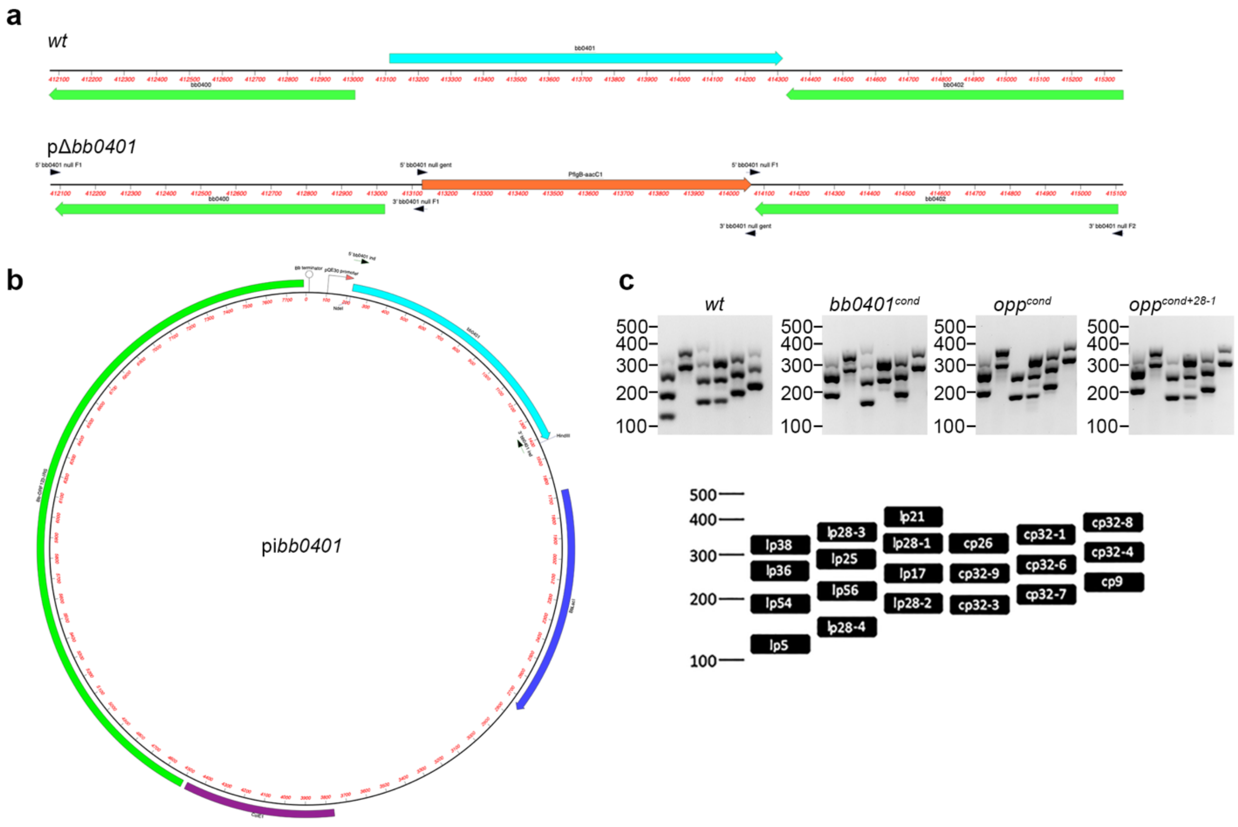

Fig S2: *bb0401* is essential for growth. a Schematic of *bb0401* locus on *B. burgdorferi* chromosome (*wt*) where gene of interest is in cyan and construction of pΔ*bb0401* where antibiotic cassette is in orange and arrows represent primer locations **b** Schematic of pi*bb0401* where gene of interest is in cyan, *lacI* in blue, *E. coli* origin of replication in purple, and cp9 shuttle vector region in green, arrows represent primer location, and restriction enzyme sites are shown. **c** Plasmid content multiplex PCRs for all strains, ladder sizes are shown in bp and a schematic of multiplex targets is displayed below.

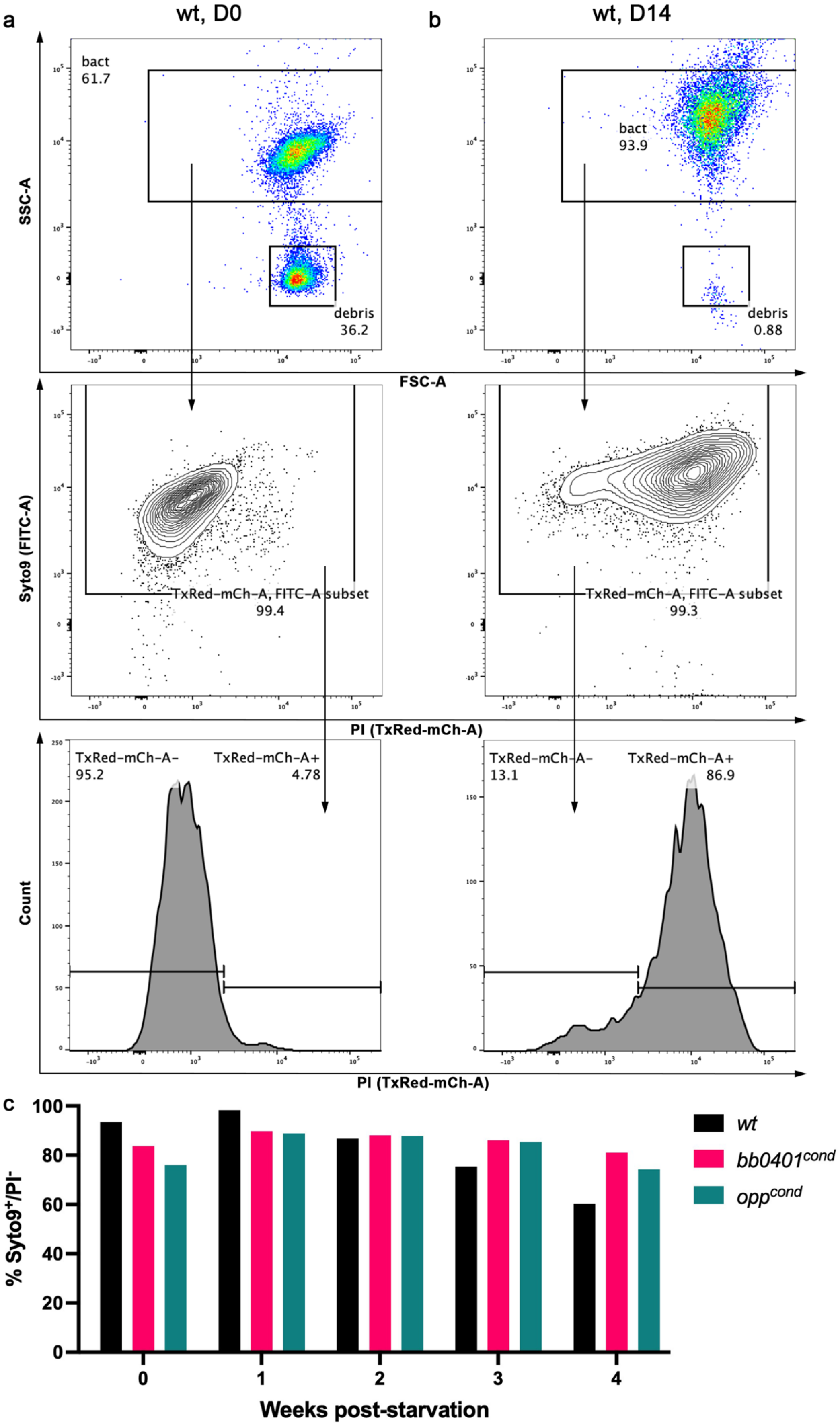

Figure S3: Amino acid transport is not essential for room temperature growth. a-b) Example gating strategy to identify live/dead cell populations using flow cytometry for *wt*. Top graph shows FSC-A/SSC-A for exclusion of debris present in the media, middle graph shows TxRed-mCh-A/FITC-A to identify the FITC-A+ subset. Bottom graph shows a histogram of TxRed-mCh-A to gate live and dead populations. c) Percent of live cells (Syto9+/PI-) during room temperature incubation sampled weekly over a four-week period. Two-way ANOVA found no statistical significance in pairwise comparisons.

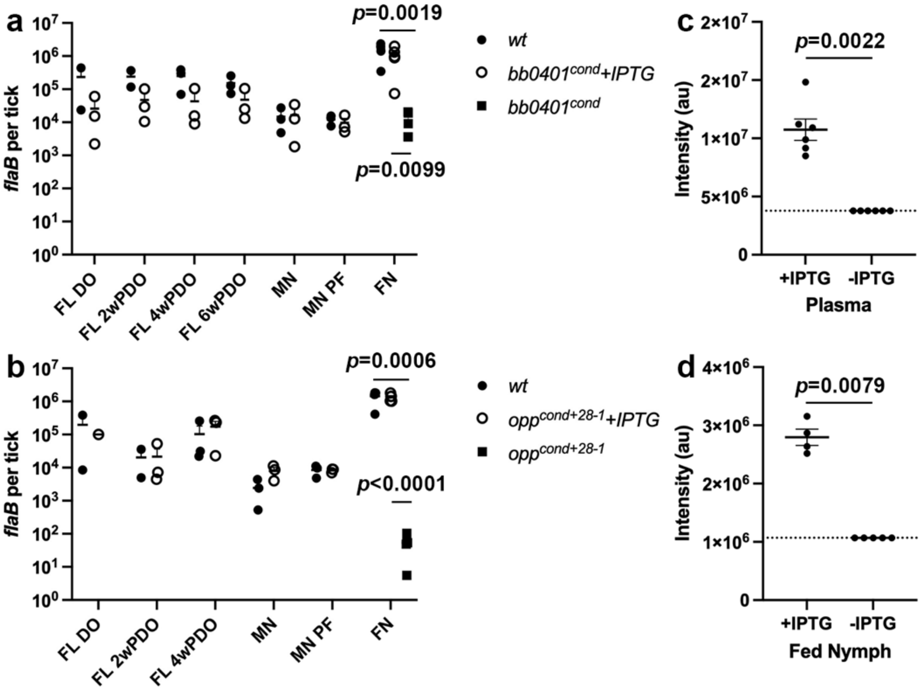

Figure S4: DNA burdens are lower in fed nymph for both mutants. Burdens as determined by qPCR of fed larvae at drop-off (FL DO), fed larvae at timepoints post-drop-off (FL #wPDO), post-molt nymphs (MN), molted nymphs prior to feeding (MN PF), and fed nymphs (FN) for a) *bb0401*^{comp} and b) *opp*$^{cond+28\text{-}1}$. IPTG detection in c) pooled mouse plasma and d) pooled fed nymphs. Dotted lined represent the LOQ. *p*-values were determined for pairwise comparisons using a two-tailed unpaired *t* test.

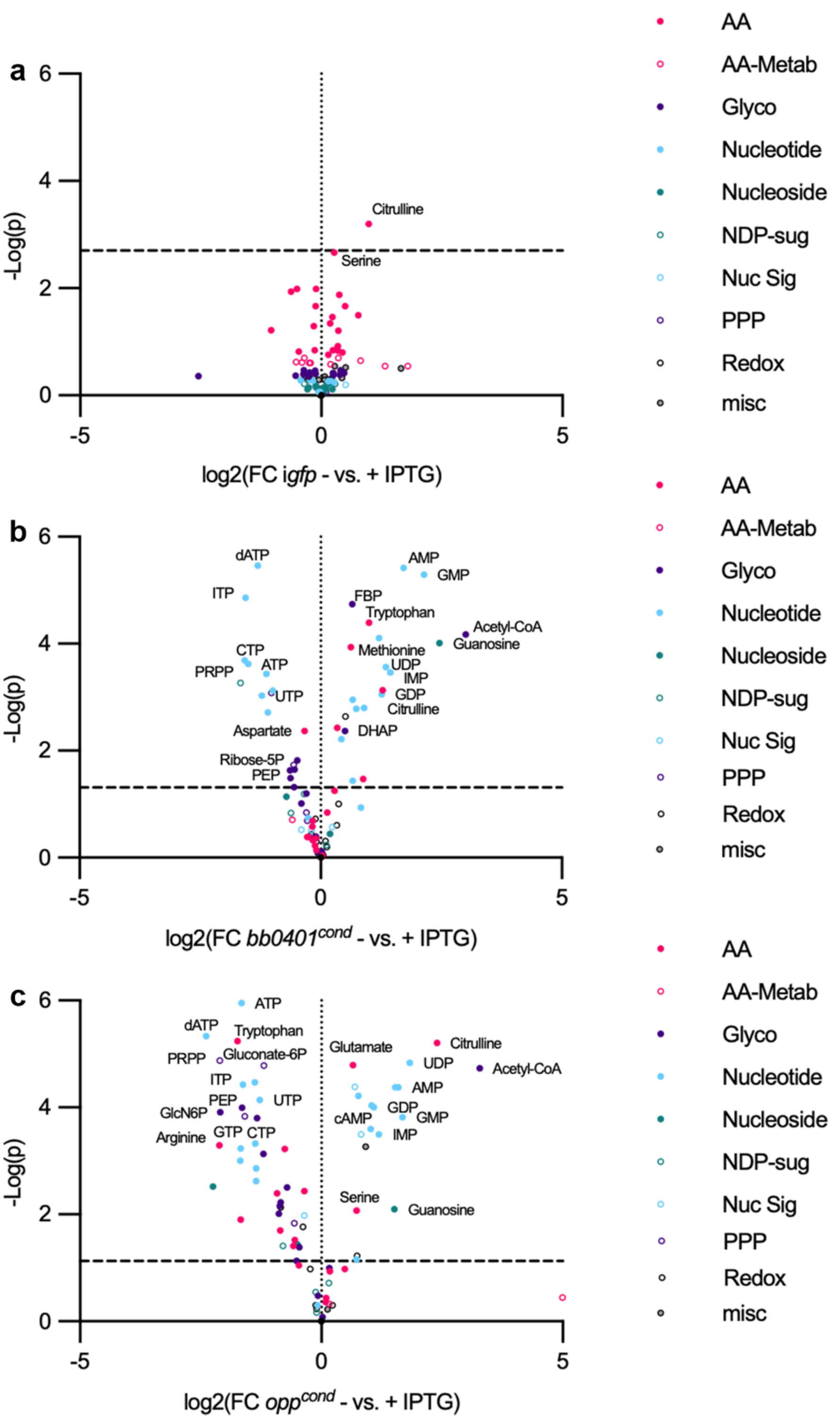

Figure S5: *opp*^{cond} starvation results is a larger metabolite shift than *bb0401*^{cond}. Volcano plots showing metabolite changes in a) i*gfp* control, b) *bb0401*^{cond}, and c) *opp*^{cond} when growth without and with 1 mM IPTG. Metabolites are color-coded by primary pathway. Dotted line represents 10% FDR. Tabulated results can be found in Table S3. AA=amino acids, AA-Metab=amino acid metabolites, Glyco=glycolysis and other carbohydrate, NDP-sug=nucleotide diphosphate sugar conjugates, Nuc Sig=signaling nucelotides, PPP=pentose phosphate pathway, Redox=redox cofactors.

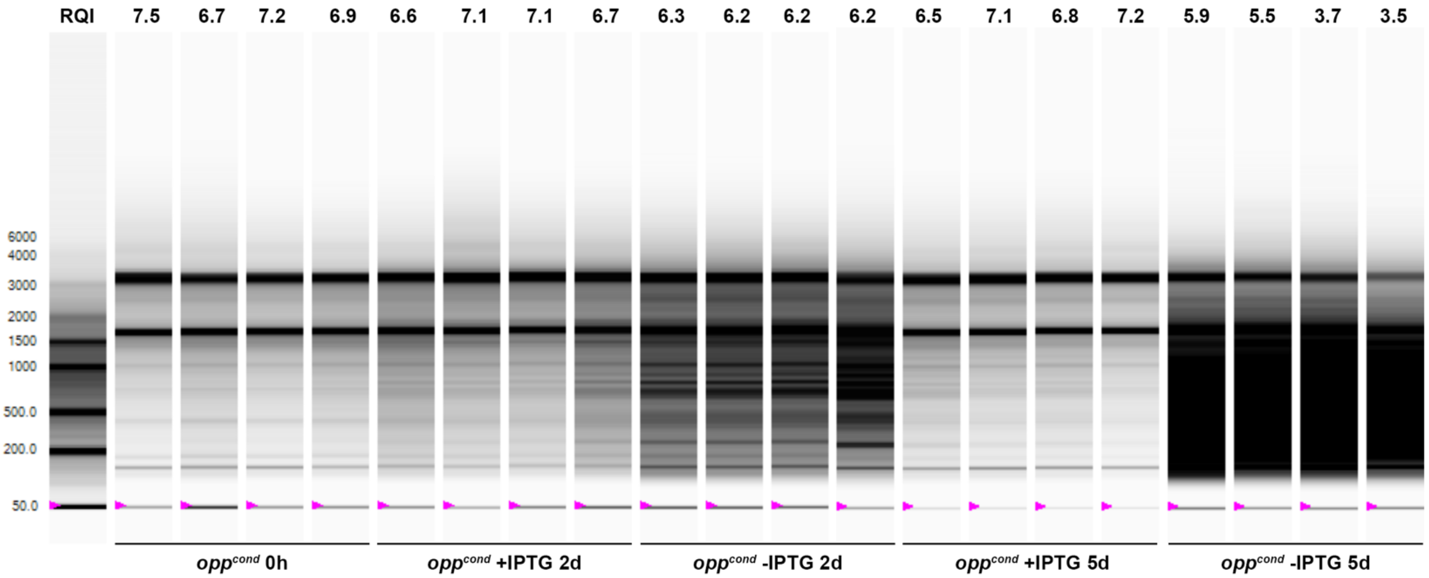

Figure S6: Prolonged starvation of *opp^{cond}* results in RNA degradation. RNA samples collected from *opp^{cond}* at timepoint 0 and without or with 1 mM IPTG at 2 d and 5 d post-incubation with their reported RNA quality indicator (RQI).

Supplementary Table 1. *Borrelia burgdorferi* strains and plasmids used in this study

Strain/Plasmid	Description	Antibiotic Resistance	Reference
B. burgdorferi			
BbG100	Wild-type strain B31 5A18 NP1 (*wt*)	Kan	[1]
BbG141A	B31 5A18 NP1 i*bb0334-35* Δ*bb0334-35* (opp^{cond})	Kan/Strep/Erm	[2]
BbG141B	B31 5A18 NP1 i*bb0334-35* Δ*bb0334-35* ($opp^{cond+lp28\text{-}1}$)	Kan/Strep/Erm	This study
BbAG320	B31 5A18 NP1 i*bb0401* (i*bb0401*)	Kan/Strep	This study
BbG119	B31 5A18 NP1 i*bb0401* Δ*bb0401* ($bb0401^{cond}$)	Kan/Strep	This study
BbP1781	B31 5A4	N/A	[3]
BbAG367	B31 5A4 i*gfp* (i*gfp*)	Strep	This study
E. coli			
Top10	*F–mcrA Δ(mrr-hsdRMS-mcrBC) φ80lacZΔM15 ΔlacX74 recA1 araD139 Δ(ara-leu)7697 galU galK λ–rpsL(StrR) endA1 nupG*	N/A	Invitrogen
Stellar	*F–, endA1, supE44, thi-1, recA1, relA1, gyrA96, phoA, Φ80d lacZΔ M15, Δ (lacZYA - argF) U169, Δ (mrr - hsdRMS - mcrBC), ΔmcrA, λ–*	N/A	Clonetech
Plasmids			
pUC19	Cloning vector	Amp	Invitrogen
pJSB275	Shuttle vector with IPTG-inducible luciferase lacking NdeI in the resistance marker	Spec/Strep	[2]
pBRV2	Gent marker	Gent	[4]
pEcAG286	pi*bb0401*	Strep	This study
pEcAG259	pΔ*bb0401*	Gent/Amp	This study
pCE320	*gfp*	Zeo	[5]
EcAG304	pi*gfp*	Strep	This study

Supplementary Table 2. Oligonucleotide primers used in this study

Designation	Sequence (5'-3')	Purpose	Reference
M13 F	CAGGAAACAGCTATGAC	Sequencing	Invitrogen
M13 R	GTAAAACGACGGCCAG	Sequencing	Invitrogen
pless Strep F	ATGAGGGAAGCGGTGATCGCCGA	Diagnostic PCR	[4]
pless Strep R	TTATTTGCCGACTACCTTGGTG	Diagnostic PCR	[4]
pless Gent F	ATGTTACGCAGCAGCAACGATG	Diagnostic PCR	[4]
pless Gent R	TTAGGTGGCGGTACTTGGGTCCA	Diagnostic PCR	[4]
5' pJSB275 seq	GATTCAATTGTGAGCGGAATAACA	Sequencing	[6]
3' pJSB275 seq	ATGCGCTTAACGGTAAAATCCAAGG	Sequencing	[6]
5' bb0401 ind	**GGAGAAATTA*CATATG***AATATAAAAATCAATTTTTTTTTCACTTTG	i*bb0401*cloning	This study
3' bb0401 ind	**CTCTATCTTC*AAGCTT***TTAATTAATTTTTTCTTGATCTTTTAATTCTTTG	i*bb0401*cloning	This study
5' bb0401 null F1	**CGACTCTAGA*GGATCC***GCCTCTTGGCCCTATC	Δ*bb0401* cloning	This study
3' bb0401 null F1	**TTGAAGCTCGGGTAG**ATGACTTCTCCTTTCAGAGATTTA	Δ*bb0401* cloning	This study
5' bb0401 null gent	**GAAAGGAGAAGTCAT**CTACCCGAGCTTCAAGG	Δ*bb0401* cloning	This study
3' bb0401 null gent	**TTAATTTGTTTAGCT**GGCGGTACTTGGGTC	Δ*bb0401* cloning	This study
5' bb0401 null F2	**GACCCAAGTACCGCC**AGCTAAACAAATTAATAGGATTTGGCA	Δ*bb0401* cloning	This study
3' bb0401 null F2	**CGGTACCCGG*GGATCC***GGCCTTTTTTGCGCAC	Δ*bb0401* cloning	This study
5' iGFP	**AAGAGGAGAAATTA*CAT****ATG*AGTAAAGGAGAAGAACTTTTC	i*gfp* cloning	This study
3' iGFP	**CTCTATCTTC*AAGCTT***TTATTTGTATAGTTCATCCATGCC	i*gfp* cloning	This study

Bold denotes overlap sequence for InFusion cloning. Italics denotes restriction sites.

References

1. Kawabata, H., S.J. Norris, and H. Watanabe, *BBE02 disruption mutants of Borrelia burgdorferi B31 have a highly transformable, infectious phenotype.* Infect Immun, 2004. **72**(12): p. 7147-54.
2. Groshong, A.M., et al., *Peptide Uptake Is Essential for Borrelia burgdorferi Viability and Involves Structural and Regulatory Complexity of its Oligopeptide Transporter.* mBio, 2017. **8**(6).
3. Purser, J.E. and S.J. Norris, *Correlation between plasmid content and infectivity in Borrelia burgdorferi.* Proc Natl Acad Sci U S A, 2000. **97**(25): p. 13865-70.
4. Caimano, M.J., et al., *The RpoS Gatekeeper in Borrelia burgdorferi: An Invariant Regulatory Scheme That Promotes Spirochete Persistence in Reservoir Hosts and Niche Diversity.* Front Microbiol, 2019. **10**: p. 1923.
5. Eggers, C.H., et al., *Identification of loci critical for replication and compatibility of a Borrelia burgdorferi cp32 plasmid and use of a cp32-based shuttle vector for the expression of fluorescent reporters in the lyme disease spirochaete.* Mol Microbiol, 2002. **43**(2): p. 281-95.
6. Groshong, A.M., M.A. McLain, and J.D. Radolf, *Host-specific functional compartmentalization within the oligopeptide transporter during the Borrelia burgdorferi enzootic cycle.* PLoS Pathog, 2021. **17**(1): p. e1009180.
